# Genetic drivers of liver cirrhosis: The role of *SERPINA1* and *PNPLA3* variants in disease onset and progression

**DOI:** 10.1371/journal.pone.0333051

**Published:** 2025-09-26

**Authors:** Mikolas Holinka, Sona Frankova, Svetlana Adamcova Selcanova, Lubomir Skladany, Ondrej Fabian, Martin Kveton, Dusan Merta, Vera Adamkova, Jaroslav A. Hubacek, Veronika Pitova, Sarka Vesela, Tomas Hucl, Milan Jirsa, Jan Sperl

**Affiliations:** 1 Department of Hepatogastroenterology, Institute for Clinical and Experimental Medicine, Prague, Czech Republic; 2 Third Faculty of Medicine, Charles University, Prague, Czech Republic; 3 HEGITO—Department of Hepatology, Gastroenterology and Liver Transplantation of 2nd Department of Internal Medicine, F. D. Roosevelt University Hospital, Banska Bystrica, Slovakia; 4 2nd Department of Internal Medicine, L. Pasteur University Hospital and P. J. Safarik University, Kosice, Slovakia; 5 Clinical and Transplant Pathology Centre, Institute for Clinical and Experimental Medicine, Prague, Czech Republic; 6 Department of Pathology and Molecular Medicine, Third Faculty of Medicine, Charles University and Thomayer Hospital, Prague, Czech Republic; 7 Anaesthesiology and Resuscitation Department, Institute for Clinical and Experimental Medicine, Prague, Czech Republic; 8 First Faculty of Medicine, Charles University, Prague, Czech Republic; 9 Preventive Cardiology Centre, Institute for Clinical and Experimental Medicine, Prague, Czech Republic; 10 Atherosclerosis Research Laboratory, Institute for Clinical and Experimental Medicine, Prague, Czech Republic; 11 Laboratory of Experimental Hepatology, Experimental Medicine Centre, Institute for Clinical and Experimental Medicine, Prague, Czech Republic; Medizinische Fakultat der RWTH Aachen, GERMANY

## Abstract

*SERPINA1 Z* and *PNPLA3* G alleles are the most potent genetic risk modifiers in chronic liver disease (CLD) progression. We aimed to test the impact of concomitant carriage of these variants on the progression of CLDs of various aetiology. The cirrhosis cohort included 1583 individuals with CLD, evaluated as candidates for liver transplantation (LTx), with alcoholic-related liver disease (ALD), metabolic dysfunction-associated (MASLD), viral (VIR), autoimmune/cholestatic (AIH-CHOL), and metabolic conditions (MET). This cohort was compared to a control population of 3483 healthy individuals. The frequency of *SERPINA1* MZ heterozygotes was significantly higher (p < 0.0001) in the entire cirrhosis group (84/1583; 5.3%) than in controls (89/3483; 2.6%), OR 2.57 (95% CI 1.92–3.44). The frequency of *SERPINA1* MZ heterozygotes was significantly higher in the subgroups with ALD and MASLD (37/557; 6.4% and 23/208; 11.1%, respectively, p < 0.0001); the frequency in the subgroups VIR, AIH-CHOL and MET did not differ from controls. The frequency of the *PNPLA3* G allele was significantly higher (p < 0.0001) in the entire cirrhosis group (880/1,583; 55.6%) than in controls (1418/3402; 41.6%); OR 1.48 (95% CI 1.36–1.99). The G allele frequency was significantly higher only in ALD, MASLD and VIR subgroups (392/577, 67.9%; 133/208, 63.9% and 139/264, 52.7%; p < 0.0001, 0.0001 and 0.0005, respectively). The frequency of the *PNPLA3* G allele was the same in cirrhotic patients carrying *SERPINA1* MM and MZ genotypes (824/1483, 55.6% vs 50/84, 59.2%, N.S.). *SERPINA1* MZ heterozygotes with ALD and MASLD were significantly younger (56.9 vs 60 years, p = 0.046) and had a higher MELD score (17 vs 15 points, p = 0.0003) at waitlisting, whereas the *PNPLA3* genotype had no impact on the age and MELD score at waitlisting. We conclude that both variant alleles increase the risk of liver cirrhosis in ALD and MASLD; however, *SERPINA1* MZ heterozygotes have more progressive chronic liver disease and need LT at a younger age.

## Introduction

Alpha-1 antitrypsin (AAT), a key acute-phase protein and the principal inhibitor of plasma serine proteases (Pi), is encoded by the *SERPINA1* gene [1]. It is primarily synthesised in hepatocytes and subsequently released into the bloodstream. Its main physiological role is to protect pulmonary tissue from the destructive activity of neutrophil elastase. The most common variant of the gene, the M allele, exists in several subtypes (M1A, M1V, M2, M3, and M4), and the MM genotype is observed in around 88% of the general population. Disease-associated alleles can be classified into three categories, with the “storage” alleles being the most relevant. This group includes the relatively prevalent Z allele (Glu342Lys, rs28929474) and two rarer mutations: Mmalton (Δ52Phe, rs775982338) and Siiyama (Ser52Phe, rs55819880) [2–4]. Proteins produced from these storage alleles are subject to abnormal intracellular processing in hepatocytes—approximately 70% are degraded within the cell, 15% are secreted, and another 15% form insoluble polymers [3,5,6]. Only a minority of these polymers are cleared, while the majority accumulate in the endoplasmic reticulum (ER). These retained aggregates can be identified by Periodic Acid-Schiff staining after diastase digestion (PASD-positive) [6]. The intracytoplasmic accumulation of AAT polymers impairs ER function and induces proteotoxic stress, mechanisms that are thought to contribute to hepatic steatosis and the progression of liver fibrosis [7].

The null alleles (Q0) are characterised by the absence of protein expression or a truncated, non-polymerising protein synthesis and represent the second subtype of AAT variant alleles.

The third type of variant allele is the S allele (Glu264Val, rs17580). It represents the most common variant allele in the Caucasian population. It is characterised by the synthesis of the dysfunctional protein undergoing partial intracellular degradation [8]. The null and/or S allele carriage is not associated with liver injury, but the Z allele carriage is. The lung emphysema and liver cirrhosis are well-described complications in ZZ homozygotes [9]. Based on a cross-sectional biopsy study and a large European study using non-invasive methods of liver fibrosis assessment, 35% and 20–36% of Pi*ZZ homozygotes develop significant liver fibrosis [10,11].

The Pi*MZ *SERPINA1* carriers have long been considered healthy. Based on recently published genetic studies, Pi*MZ heterozygotes also present with an increased risk of liver fibrosis and cirrhosis, especially if they suffer from another chronic liver disease of a different aetiology [12]. The precipitated Z-protein aggregates in the hepatocytes of Pi*MZ heterozygotes may also contribute to a more severe course of liver disease [13].

The MZ genotype carriage turned out to be the strongest genetic factor of liver cirrhosis development in the group of patients with metabolic dysfunction-associated steatotic liver disease (MASLD) and in people presenting with harmful drinking. The risk was higher in MZ heterozygotes than in the carriers of variant alleles of liver disease-modifying genes, such as *PNPLA3, TM6SF2* and *MBOAT7* [14,15]. The carriage of the Pi*MZ genotype was finally validated in two large population studies [14,15].

In Hakim’s study [14], the authors assessed the genetic interplay between the variants in *PNPLA3*, *TM6SF2*, and *HSD17B13,* and they did not prove any epistasis between *SERPINA1* Z allele carriage of the above-mentioned variants regarding higher AST and ALT serum activities. In a mouse experiment, Volkert et al. demonstrated that the carriage of the *SERPINA1* Z allele was protective against liver damage and obesity in the mice fed with a Western-type diet [16].

In our previously published study [17], which focused on patients with advanced liver cirrhosis, we described that the Pi*MZ heterozygotes presented with a more rapid progression of liver disease over time. They needed liver transplantation at a younger age, and at the time of waitlisting, they presented with a higher MELD score than their Pi*MM counterparts [17].

*PNPLA3* rs738409 is a major genetic determinant of steatotic liver disease and its severity [18,19]. The wild-type protein contributes to maintaining lipid homeostasis by balancing triglyceride turnover, whereas the variant protein impairs the enzyme’s lipolytic activity, leading to the accumulation of triglycerides within hepatocytes. *PNPLA3* expression is highly regulated by changes in energy balance [20] and dietary factors [21]. Lifestyle and environmental factors can exacerbate or attenuate the inherited risk of liver disease and mortality: it has recently been suggested that a higher BMI and high-risk consumption of alcohol may significantly increase the risk of fatty liver and advanced fibrosis among carriers of the unfavourable *PNPLA3* genotypes [22].

As the *PNPLA3* variant allele is the most frequent risk modifier among cirrhotic patients, we analysed a larger group of cirrhotic patients to establish the impact of concomitant carriage of both variants on the severity of liver disease in the liver transplant settings.

## Methods

### Study design

The cirrhosis group (cases) included 1,583 liver transplant (LT) candidates with chronic advanced liver disease undergoing their pre-transplant work-up in two LT centres, the Institute for Clinical and Experimental Medicine, Prague, Czech Republic, and Banska Bystrica, Slovakia, between July 1, 1994, and December 31, 2024. The patients undergoing LT owing to aetiology of liver disease other than cirrhosis were excluded from the study (acute liver failure, polycystic liver disease, tumours in non-cirrhotic liver, etc.).

The patients were considered and enlisted for LT owing to chronic advanced liver disease, i.e., liver cirrhosis, using standard criteria for the evaluation of liver dysfunction, the MELD and Child-Pugh’s score). In 343 patients, a small hepatocellular carcinoma in the cirrhotic liver represented the leading indication for LT. In all patients, the diagnosis of liver cirrhosis and HCC was confirmed in the liver explant using standard histological techniques.

Patients’ demographic, clinical and laboratory data were extracted from the hospital information systems and included the values at the time of waiting list enrolment (Table 1). The data were retrospectively analysed and accessed between January 5 and January 26, 2025, by the authors (S.F., M.H., S.A.S. and D.M.).

**Table 1:** Patients’ characteristics according to *SERPINA1* genotypes.

	Total MM + MZ (1567)	MM (1483)	MZ (84)	P value (MM vs MZ)
Age, years	56.6 (18–76.1)	56.7 (18–76.1)	55.2 (21.8–74.8)	0.74
BMI, kg/m^2^	25.6 (14.8–45.1)	25.5 (14.8–45.1)	27.5 (18.1–43.0)	**0.02**
MELD score	15 (6–40)	14 (6–40)	16.5 (9–34)	**< 0.0001**
Serum A1AT, g/L	1.6 (0.35–3.5)	1.63 (0.68–6.0)	0.91 (0.35–2.05)	**< 0.0001**
Serum Creatinine, μmol/L	73.0 (20.2–699)	72.9 (20.2–699)	76.2 (28–565)	0.2
Serum Bilirubin, μmol/L	45.2 (4.3–1164)	44.4 (4.3–1164)	59.0 (6.8–493.3)	0.06
Prothrombin time, INR	1.36 (0.6–6.9)	1.34 (0.8–6.0)	1.49 (0.6–3.3)	**< 0.0001**
Serum Albumin, g/L	29.5 (9.8–53.3)	29.7 (9.8–53.3)	24.9 (15.7–44.6)	**< 0.0001**
Aetiology of liver cirrhosis:				
ALD	577	540 (93.6%)	37 (6.4%)	N.A.
MASLD	208	185 (88.9%)	23 (11.1%)	
VIR	264	252 (95.5%)	12 (4.5%)	
AIH-CHOL	443	432 (97.5%)	11 (2.5%)	
MET*	91	74 (81.3%)	1 (1.1%)	

ALD, alcoholic liver disease; MASLD, metabolic dysfunction-associated steatotic liver disease; AIH-CHOL, autoimmune and cholestatic liver disease; VIR, chronic viral hepatitis; MET, metabolic liver disease; N.A., not applicable. *The group also included 16 ZZ *SERPINA1* homozygotes.

The study cohort was categorised into five subgroups based on the aetiology of cirrhosis: alcohol-related liver disease (ALD, 577 individuals), metabolic dysfunction-associated steatotic liver disease (MASLD, 208 individuals), autoimmune and cholestatic liver disorders (AIH-CHOL, 443 individuals), chronic viral hepatitis (VIR, 264 individuals), and inherited metabolic liver disorders (MET, 91 individuals). Group assignment was determined using the patient’s clinical history, documented alcohol consumption, and diagnostic data collected during the liver transplant evaluation. The AIH-CHOL group comprised cases of autoimmune hepatitis, primary biliary cholangitis, primary sclerosing cholangitis, and variant syndromes. The VIR group included patients with chronic hepatitis B, hepatitis C, or hepatitis B/D coinfections. The MET subgroup encompassed individuals with Wilson’s disease, hereditary hemochromatosis, and homozygous ZZ variants of the *SERPINA1* gene. Including ZZ homozygotes enabled the comparison of genotype distribution across both patient and control groups.

Genotypic data for *SERPINA1* and *PNPLA3* in the study cohort were compared with those of 3402 healthy controls. These controls were drawn from the Czech MONICA population study, which focused on cardiovascular and hypertension risk assessment in the general population [23]. All participants, both in the patient cohort and control group, were of Caucasian descent.

### Human DNA genotyping

Genomic DNA was extracted from peripheral blood samples using the QIAamp DNA Blood Mini Kit (Qiagen, Hilden, Germany). Genotyping of the *SERPINA1* rs28929474 (PI*Z) variant was conducted using the TaqMan SNP Genotyping Assay C_34508510_10, and the rs17580 (PI*S) variant using Assay C_594695_20 (Thermo Fisher Scientific, Waltham, MA, USA). In addition, all patients were genotyped for the *PNPLA3* rs738409 c.444C>G polymorphism using the TaqMan Predesigned SNP Genotyping Assay C_7241_10. All assays followed the manufacturer’s instructions using the ABI 7300 Real-Time PCR System and the QuantStudio 6 Flex Real-Time PCR System (Thermo Fisher Scientific).

### AAT serum concentration assessment

The concentrations of serum AAT were assessed in the study subjects in the pre-transplant work-up using immunoturbidimetry in the local biochemistry laboratories. The normal range for AAT was 0.88–1.74 g/L.

### Histological analysis of the explanted liver tissue

We assessed the histopathology samples from the explanted livers. From each explanted liver, one representative paraffin block from the left and one from the right liver lobe were selected and processed for histopathological examination. The examination included haematoxylin and eosin (HE), Periodic Acid-Schiff diastase (PASD) stains, and immunohistochemistry.

HE was performed on three µm-thick sections, while PASD staining and immunohistochemistry were performed on 4 µm sections. The PASD-positive intrahepatic aggregates were assessed and graded semiquantitatively based on their density, extent, and distribution.

Antigen retrieval was conducted in the BenchMark ULTRA PLUS instrument using the heat-induced epitope retrieval (HIER) technique with Cell Conditioning 1 solution (Ventana Medical Systems, Inc.; catalogue number: 950-124). Immunohistochemical staining was performed using a prediluted polyclonal rabbit anti-AAT primary antibody (Ventana Medical Systems, Inc.; catalogue number: 760-2605). Detection of bound primary antibodies was achieved using the ultraView Universal DAB Detection Kit (Ventana Medical Systems, Inc.; catalogue number: 760-500), which included a secondary antibody.

Aggregate density and the extent were evaluated according to Clark et al. [10] as described in our previous study [17]. Furthermore, we assessed the typical periseptal distribution of the aggregates in the cirrhotic livers.

### Statistical analysis

Statistical analysis was performed using GraphPad Prism version 10.3.1 for Mac, GraphPad Software, San Diego, California, USA (www.graphpad.com) and R programming language version 4.4.2 (www.r-project.org).

Clinical characteristics were analysed in a descriptive way and reported as means and standard deviations or medians and ranges where the assumptions of normal distribution were not met. The categorical variables were expressed as frequencies (%). Categorical data were analysed using the chi-square test. For continuous data, Student’s t-test and one-way ANOVA or the non-parametric Mann-Whitney and Kruskal-Wallis tests were used appropriately. Testing for genetic associations was performed as described in [24]. Risk factors were examined using multivariate logistic regression analysis.

All statistical analyses were two-sided, and the P value of < 0.05 was considered statistically significant throughout the study.

### Ethics statement

This study was approved by the Ethics Committee of the Institute for Clinical and Experimental Medicine and the Thomayer University Hospital, Prague, Czech Republic, with study approval number G-21-55. The study was carried out in accordance with the Declaration of Helsinki. All study participants gave written consent, including genetic testing, to the storage of blood samples and agreed to use blood for future research. The written consent was obtained before enlistment for LT.

## Results

### Genotype frequencies of *SERPINA1* and *PNPLA3* and the risk of liver cirrhosis

The frequencies of the particular genotypes are presented in Tables 2 and 3. The frequency of *SERPINA1* MZ genotype was significantly higher (p < 0.0001) in the cirrhosis group (84 of 1583; 5.3%) than in controls (89 of 3483; 2.6%). The carriage of *SERPINA1* MZ genotype increased the risk of liver cirrhosis (OR 2.57; 95% CI 1.92–3.44).

**Table 2:** *SERPINA1* genotype frequencies in cases and controls.

	Cirrhosis		Controls		P value
MM	1483	93.8%	3394	97.4%	**<0.0001** ^a^ ^,^ ^b^
MZ	84	5.2%	89	2.6%	
ZZ	16	1.0%	0	0%	
Total	1583		3483		
MS^*^	38		101		
SS^*^	0		2		

^a^Allelic model (*SERPINA1* MM vs MZ + ZZ),

^b^Recessive model (*SERPINA1* ZZ vs MZ + MM).

* The genotypes MS and SS were excluded from further evaluation because of the well-known lack of impact on the risk of liver cirrhosis.

**Table 3:** *PNPLA3* genotype frequencies in cases and controls.

	Cirrhosis		Controls		P value
CC	702	44.3%	2035	58.4%	**<0.0001** ^a^ ^,^ ^b^
CG	631	39.9%	1259	36.1%	
GG	250	15.8%	189	5.5%	
Total	1583		3483		

^a^Allelic model (*PNPLA3* CC vs CG + GG),

^b^Recessive model (*PNPLA3* GG vs CG + CC).

When evaluating the genotype frequency in the subgroups of cases according to the liver cirrhosis aetiology, the frequency of *SERPINA1* MZ genotype carriers was significantly higher in the subgroups of cases with ALD and MASLD cirrhosis (37 of 577; 6.4% and 23 of 208; 11.1%, respectively, p < 0.0001 for both groups) than in controls (89 of 3402; 2.6%). The frequency of *SERPINA1* MZ genotype did not differ when comparing the pooled subgroups VIR, AIH-CHOL and MET with controls (24 of 798; 3.0% vs 89 of 3483, 2.6%; N.S.) The *SERPINA1* MZ genotype carriage increased the risk of liver cirrhosis only in patients with ALD (OR 2.63; 95% CI 1.78–3.89) and MASLD (OR 4.74; 95% CI 2.92–7.70), p < 0.0001 for both groups.

The frequency of the *PNPLA3* G allele carriers was significantly higher (p < 0.0001) in the cirrhosis group (880 of 1583; 55.6%) than in controls (1418 of 3402; 41.6%). Carrying at least one allele G of *PNPLA3* increased the risk of liver cirrhosis (OR 1.48; 95% CI 1.36–1.99). When evaluating the genotype frequency in the subgroups of cases according to the liver cirrhosis aetiology, the frequency of *PNPLA3 G* allele was significantly higher in the subgroups with ALD, MASLD and VIR cirrhosis (392 of 577, 67.9%; 133 of 208, 63.9% and 139 of 264, 52,7% p < 0.0001 for all ALD and MET and p = 0.0005 for VIR) than in controls (1448 of 3483; 41.6%). The frequency of *PNPLA3* G allele carriage did not differ in AIH-CHOL and MET with controls (180 of 443; 40.6% and 42 of 91; 46.1%; N.S. for both groups).

The frequency of the *PNPLA3* G allele was the same in cirrhotic patients carrying *SERPINA1* MM and MZ genotypes (824 of 1483, 55.6% vs 50 of 84, 59.2%, N.S.).

### Impact of *SERPINA1* and *PNPLA3* genotypes on age at waitlisting

The median age at the waiting list enrolment in the entire group did not differ in the subgroups of *SERPINA1* genotypes (MM 56.7 years, MZ 55.2 years, N.S.). When evaluating the age only in the cirrhotic patients with ALD and MASLD together, the carriers of *SERPINA1* MZ genotype were significantly younger than MM carriers (56.9 vs 60.0 years, p = 0.046).

The concomitant carriage of different *PNPLA3* genotypes had no impact on the age at listing in either the MZ heterozygotes or the MM homozygotes group (Table 4). When calculating the median age at waitlisting according to the *PNPLA3* genotype (CC vs CG + CG) in the entire cirrhotic group, the CC genotype carriers were surprisingly younger than the CG + GG carriers (55.9 vs 58.3 years, p = 0.0002). When evaluating *PNPLA3* genotypes and age at waitlisting only in the group of the ALD and MASLD patients, the age of both groups was comparable (60.0 vs 59.7, N.S.).

**Table 4:** Age at waitlisting according to the *SERPINA1* and *PNPLA3* genotypes in the pooled ALD + MASLD group.

*SERPINA1*	*PNPLA3*	Age (years)	P value
MM	All	60.0*	*0.046
	CC	59.8	N.S.
	CG + GG	60.2	
MZ	All	56.9*	*0.046
	CC	56.5	N.S.
	CG + GG	57.2	

N.S. Not significant.

### Impact of *SERPINA1* and *PNPLA3* genotypes on liver disease severity at waitlisting

The median MELD score at the waiting list enrolment in the entire group was significantly higher in the cirrhotic patients carrying the *SERPINA1* MZ genotype than in MM homozygotes (16.5 vs 14 points, p = 0.0001). When evaluating the MELD score only in the cirrhotic patients with ALD and MASLD together, the liver dysfunction according to MELD score was even more pronounced (17 vs 15 points, p = 0.0003).

The concomitant carriage of different *PNPLA3* genotypes had no impact on the MELD score at listing in either the MZ heterozygotes or the MM homozygotes group (Table 5).

**Table 5:** MELD score at waitlisting according to the *SERPINA1* and *PNPLA3* genotypes in the pooled ALD + MASLD group.

*SERPINA1*	*PNPLA3*	MELD score (points)	P value
MM	All	15*	*0.0003
	CC	15	N.S.
	CG + GG	14	
MZ	All	17*	*0.0003
	CC	16.5	N.S.
	CG + GG	16.5	

N.S. Not significant.

When evaluating the MELD score at waitlisting according to the *PNPLA3* genotype (CC vs CG + CG) in the entire cirrhotic group, there was no difference between CC and CG + GG genotype carriers (15 vs 15 points, N.S.). The same values were obtained when evaluating *PNPLA3* genotypes and age at waitlisting only in the group of the ALD and MASLD patients (15 vs 15 points, N.S.).

### Immunohistological analysis of the explanted liver tissue

**Fig 1:**
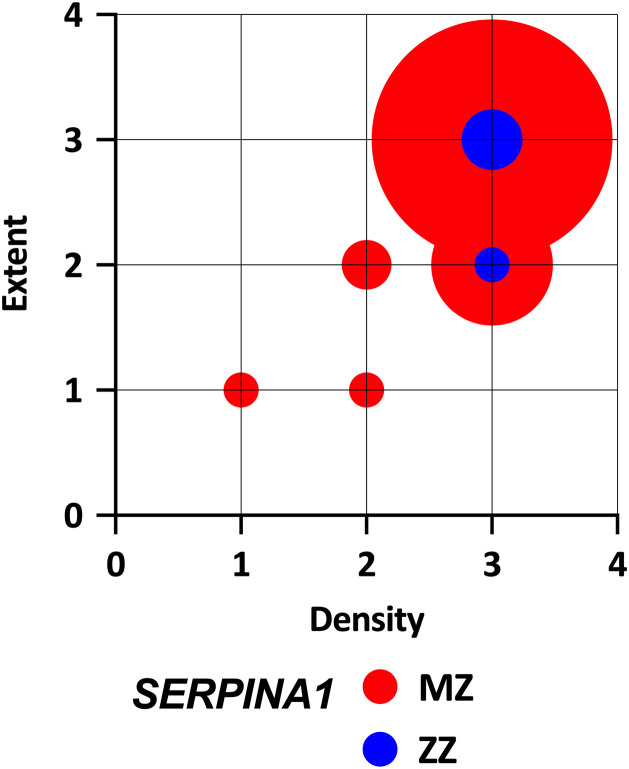
Extent and density of intrahepatic PASD positive aggregates according to *SERPINA1* genotype.

**Fig 2:**
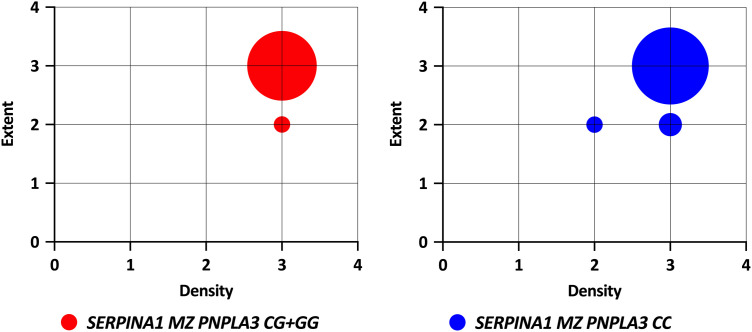
Extent and density of intrahepatic PASD positive aggregates according to *SERPINA1* and *PNPLA3* genotype.

The periseptal distribution of AAT aggregates typical of AAT deficiency disease was present in all 63 (100%) available liver explants in the *SERPINA1* MZ genotype carriers and four ZZ genotype carriers. The morphological patterns of liver samples from the ZZ homozygotes and MZ heterozygotes were identical. The semi-quantitative evaluation showed that the samples of patients with both genotypes achieved comparable scores for the extent and density of aggregates (Fig 1). Furthermore, the density and extent of precipitated AAT aggregates in *SERPINA1* MZ heterozygotes did not differ between *PNPLA3* CC and *PNPLA* CG and CG genotype carriers (Fig 2).

## Discussion

Our group of liver transplant candidates with liver cirrhosis of various aetiologies showed a significantly higher prevalence of unfavourable variants in the two studied genes, *SERPINA1* and *PNPLA3*. Specifically, there were higher frequencies of *SERPINA1* MZ heterozygotes and *PNPLA3* G allele carriers (CG and GG genotypes). Both genetic variants contributed to the initiation of chronic liver disease in their carriers.

The rate of individuals with *SERPINA1* MZ genotypes was significantly higher among the patients with cirrhosis related to harmful alcohol consumption and metabolic dysfunction-associated steatotic liver disease. Individuals with *PNPLA3* CG and GG genotypes were more frequently observed not only in these two subgroups but also among patients with virus-related liver cirrhosis. Given that both gene variants are associated with hepatic steatosis [18,25], it is highly probable that they facilitated the onset of liver diseases in which steatosis represents an initial pathological stage. On the other hand, in other aetiologies such as viral, autoimmune, or cholestatic, the dominant mechanisms of injury are immune-mediated cytotoxicity or bile acid-induced damage, in which the impaired lipid droplet metabolism plays an inferior role. Thus, *PNPLA3* or *SERPINA1* variants may not substantially modify disease progression in these settings. The late features of the liver disease in our cohort appeared to be associated only with the *SERPINA1* genotype, while no significant impact of the *PNPLA3* genotype was observed.

The amount of precipitated Z-protein in the liver of *SERPINA1* MZ heterozygotes increases with the severity of liver disease [13]. All analysed MZ heterozygotes reached levels of Z-protein precipitation comparable to those observed in ZZ homozygotes. The extent and density of the Z-protein aggregates were independent of the *PNPLA3* genotype, as semi-quantitative evaluation revealed no differences among individuals with different *PNPLA3* genotypes.

Furthermore, parameters evaluating the severity of liver disease, specifically the MELD score and patients’ age, differed significantly between subgroups of *SERPINA1* MM homozygotes and MZ heterozygotes. MZ heterozygotes were younger at the time of waitlisting but presented with significantly higher MELD scores, implying a more rapid progression of liver disease in this group.

Beyond its known interaction with neutrophil elastase, AAT binds several other proteins, including tumour necrosis factor α (TNFα). Low levels of AAT may explain the higher serum TNFα concentrations observed in MZ genotype carriers. TNFα plays a crucial role in the pathogenesis of complications of liver cirrhosis. Our previous study demonstrated that high levels of TNFα are associated with an increased risk of severe bacterial infections, which frequently trigger decompensating events in cirrhosis [26]. Elevated TNFα may also contribute to the development of circulatory dysfunction in liver cirrhosis.

The distinct roles of *SERPINA1* and *PNPLA3* variants in the initiation and progression of liver disease were previously described by Volkert et al., in both human and experimental mouse models: the *PNPLA3* polymorphism in the absence of additional metabolic risk factors is insufficient to drive the development of advanced liver disease in *SERPINA1 Z* allele carriers [16]. In our opinion, their findings regarding the role of both genes in the course of liver disease are compatible with our results and can explain the surprisingly younger age of the *PNPLA3* CC carriers in the whole cohort of cirrhotic patients.

Our allelic association study’s limitations are its retrospective design and the absence of a validation cohort. However, the study is based on robust clinical data from a strictly homogeneous, large patient population.

## Supporting information

S1 File A1AT_2025_source data_13_4_2025_PLOS_ONE_anonymised. (XLSX)
